# Zirconium Phase Transformation under Static High Pressure and ω-Zr Phase Stability at High Temperatures

**DOI:** 10.3390/ma12142244

**Published:** 2019-07-12

**Authors:** Lucyna Jaworska, Jolanta Cyboron, Slawomir Cygan, Adam Zwolinski, Boguslaw Onderka, Tomasz Skrzekut

**Affiliations:** 1 Faculty of Non-Ferrous Metals, AGH University of Science and Technology, A. Mickiewicza Av. 30, 30-059 Krakow, Poland; 2 Lukasiewicz Research Network—Institute of Advanced Manufacturing Technology, Wroclawska St. 37a, 30-011 Krakow, Poland

**Keywords:** zirconium powders, HPHT pressing and sintering, phase composition, residual stresses, stability

## Abstract

High-purity Zr has been observed to undergo a phase transformation from the α-phase to the hexagonal ω-phase under high pressure generated either statically or by shock loading. The transition pressure from α-Zr to ω-Zr at 300 K is 2.10 GPa. The main aim of this research was to determine the conditions of α-Zr in ω-Zr transformation and the state of stresses after the high-pressure pressing and sintering of zirconium powders. Commercially acquired zirconium powders of 99.9% and 98.8% purity were used in this study. Qualitative and quantitative phase analysis of the materials was carried out using X-ray diffraction. The materials were statically pressed and sintered using a Bridgman-type toroidal apparatus at under 4.0 and 7.8 GPa. After pressing, the transformation proceeded for the zirconium powder containing 98.8% purity (with hydrides admixture) but did not occur for the high-purity zirconium powders with 99.9% purity. The zirconium powders were sintered using the HPHT (High Pressure—High Temperature) method at temperatures of 1273 K and 1473 K. The transformation proceeded for both powders. The highest contribution of the ω-Zr phase was obtained in the zirconium (98.8% purity with the hydrides contents) sintered for 1 min at a temperature of 1473 K and a pressure of 7.8. The ω-phase content was 87 wt.%. The stress measurement was performed for the pressed and sintered materials using the sin^2^ψ X-ray diffraction method. The higher sintering temperature resulted in a decrease of the residual stresses in the ω-Zr phase for the sintered zirconium. The higher levels of stress limited the transformation of the α-Zr phase into the ω-Zr phase. Investigated materials characterized by higher compressive macrostresses were also typical of the greater stability of the ω-Zr phase at high temperatures.

## 1. Introduction

At ambient conditions, zirconium crystallises in a hexagonal close-packed (hcp) structure known as the α phase. At atmospheric pressure with temperature up to 1135 K, it transforms into a body-centred cubic (bcc) structure commonly referred to as the β phase. On the other hand, with the increase of pressure, α-Zr (P63/mmc) transforms into another hexagonal structure, not close-packed and named the ω phase (P6/mmm), which has three atoms per unit cell. The ω-Zr can be considered as a metastable phase in ambient conditions when pressure is released. It undergoes the reverse transformation to the α phase at temperatures above 470 K [1,2]. The phase transition from α-Zr to β-Zr at atmospheric pressure occurs at 1135 K. The transition pressure from α-Zr to ω-Zr is estimated as 2.10 GPa at 300 K and the predicted triple point has the coordinates 6.35 GPa and 910 K [3,4]. The pressure-induced transformation in (hcp) structures was reported to proceed via two different paths: a direct path—α→ω, and an indirect path—α→β→ω [5].

High-purity Zr was observed to undergo a phase transformation from the α-phase to the hexagonal ω-phase (c/a = 0.623) under high pressure generated either statically or by shock loading [6,7,8,9]. Zirconium was also studied under static pressure by Bridgman [10], who reported a resistance anomaly, indicating the α zirconium phase transition into ω zirconium at 5.9 GPa. Rabinkin et al. [11] observed the omega phase in pure Zr formed under a high pressure of 6.0 GPa at 300 K. Pressurized Zr had a two-phase structure consisting of small elongated ellipsoidal ω particles which were precipitated in an α matrix. Usually, the ω-phase is obtained by applying hydrostatic pressure (diamond anvil cell) DIA or D-DIA apparatus [12,13,14]. DIA is a cubic-diamond anvil apparatus and D-DIA is a type of multi-anvil deformation apparatus.

Cerreta et al. [9] showed that the ω-Zr phase begins to form above 7 GPa under dynamic conditions. Specifically, upon shock-loading of Zr to stresses above 7 GPa and then unloading to ambient pressure, soft-recovered specimens were observed to retain high fractions of metastable ω phase, as much as 80 vol.%. The ω-Zr phase was also observed in some ultra-rapid quenched zirconium-base alloys (Zr-Cu, Zr-Ni, Zr-Co, Zr-Fe) with more than 2.5 at.% of solute additions [15]. Pichon et al. [16] detected the ω-Zr phase in thin films obtained by ion beam sputtering.

The pathway for the α to ω change (or the reverse transformation) is not well understood [8,11,17]. Under both quasi-static and dynamic loading rates, hexagonal close-packed (hcp) zirconium is known to deform through a combination of a slip and a twinning. Most previous studies on pure hcp metals focused on the pressure-induced α→ω phase transition proceeded within titanium and zirconium. This phase transition is believed to be a diffusionless displacive transformation. The pressure of the α→ω phase transition increases with the rise of the interstitial content of zirconium lattice. Crystallographically, large interstitial elements, such as oxygen, occupy the space within the lattice, which is critical for the phase transformation [9,11]. Results obtained by Cerreta et al. [9] indicated that plastic deformation and the volume fraction of the high pressure phase play important roles in determining the subsequent material properties. The role of the retained ω phase in enhanced hardening was observed from the hardness data and from the stress-strain data for the retained ω phase of 80% and 0%. An increasing volume fraction of the retained ω phase led to enhanced hardening. This observation shows ω-phase Zr to be more resistant to deformation than α-phase Zr [18].

The composite microstructure of two-phase (α/ω) shock-treated zirconium was studied in situ during heating (constant heating rate and isothermally) with high-energy X-ray diffraction techniques. Stresses in the α-Zr were estimated to be a superposition of a hydrostatic component (of order 50 MPa) and a uniaxial component (of order 600 MPa) along the *c*-axis. These stresses were relaxed during the reverse transformation [2]. In situ X-ray diffraction experiments were completed in order to better understand the stability of two-phase (α/ω) shocked Zr microstructures. Presumably, microstructural elements prevent the system from reaching equilibrium and completing the reverse transformation from the high-pressure ω phase to the stable α phase [19].

The main aim of the current research was to determine the conditions of α-Zr to ω-Zr transformation and the state of stress after high-pressure pressing and sintering of zirconium powders. 

## 2. Materials and Methods

Commercially produced high-purity zirconium powders from two different sources were used in this study. The characteristics of the purity and particle sizes of these powders are presented in Table 1.

**Figure 1:**
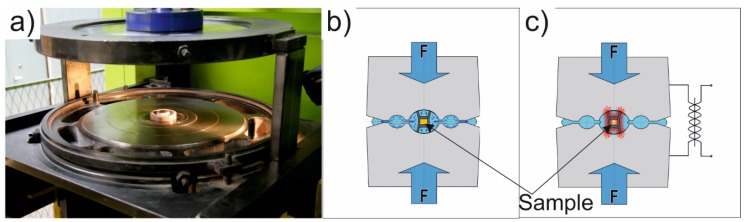
The Bridgman type toroidal HPHT anvils with an insert (**a**); cross-section scheme of the anvils during compression (**b**) cross-section scheme of the anvils during sintering (**c**).

Compressed powders and sintered discs were used for the tests. The Zr 1 and Zr 2 powders were pressed at 90 MPa into cylindrical shapes with 15 mm diameters. The green discs were placed into the internal graphite heater in a special pyrophyllite gasket assembly for the high-pressure pressing and sintering. The shapes of the anvils and pyrophillite gaskets provided pseudoisostatic pressure conditions in the compacted material. The experiment was carried out using high pressure and high temperature (HPHT) of the Bridgman type toroidal apparatus, designed for sintering at temperatures of 1273 ± 50 K and 1473 ± 50 K, as well as pressures of 4 GPa and 7.8 GPa. In this study, the applied pressure of 4 GPa was the lowest pressure possible obtained by the apparatus, while 7.8 GPa was the upper limit of the apparatus capacity. The Bridgman type HPHT (toroidal) anvils and a scheme of its cross-section are presented in Figure 1.

The XRD (X-Ray Diffraction) phase and residual-stress analysis of the obtained material was carried out by an X-ray diffraction method with the use of a PANalytical EMPYREAN diffractometer (Panalytical, Almelo, The Netherlands) with a copper radiation (λCu Kα = 1.5418 Å), a PIXcel counter and a nickel filter. The quantitative phase analysis and the refinement of the unit cell parameters for the studied materials were performed using the High Score Plus 4.0 PANalytical software based on the Rietveld refinement [20] and the ICDD (PDF-4 + 2018) data files. A phase composition analysis in the high temperature range was made in a high-temperature chamber (Anton Paar HTK 2000N, Almelo, The Netherlands). The measurement procedure consisted of 13 steps and every step consisted of heating at a rate of 293 K/min, a 10-min temperature stabilization period, and a 30-min measurement. All the measurements were conducted in vacuum ambient (1 × 10^−3^ Pa). The measurements started at the room temperature (RT) of 298 K, and next, data were recorded from 373 K up to 1473 K every 100 K. Macroscopic residual stresses were determined from a α-Zr_(203)_ diffraction line and a ω-Zr_(311)_ one. The spectra for the stress measurements where obtained with 0.02° of 2θ step and a 800 s acquisition time in the range of 88.5°–93.5° for α-Zr_(203)_ and 84.5°–87.5° for ω-Zr_(311)_. The registered intensities were fitted using a modified Lorentzian profile (fitting a Pearson VII function with a fixed shape parameter m = 2.0).

## 3. Results and Discussion

### 3.1. Phase Transition during High-Pressure Compacting and Sintering

**Figure 2:**
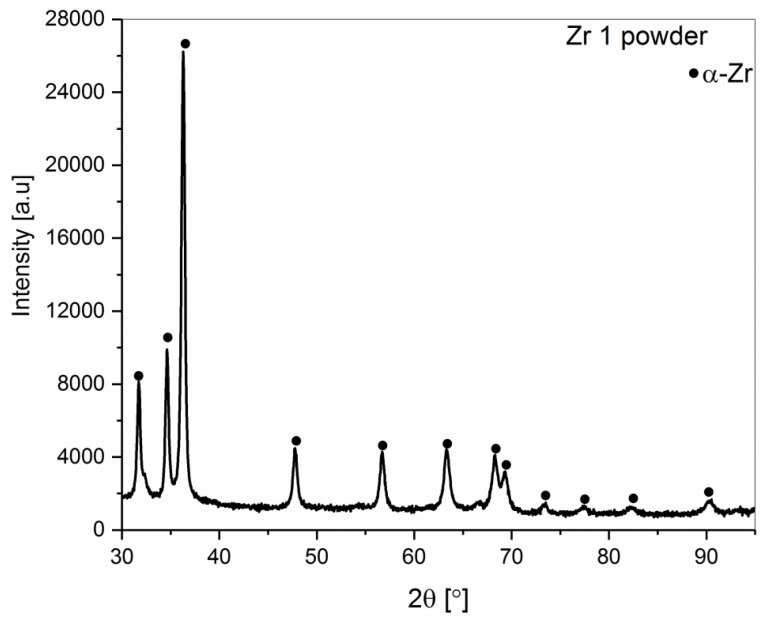
The XRD pattern of the Zr 1 powder (99.9% purity).

**Figure 3:**
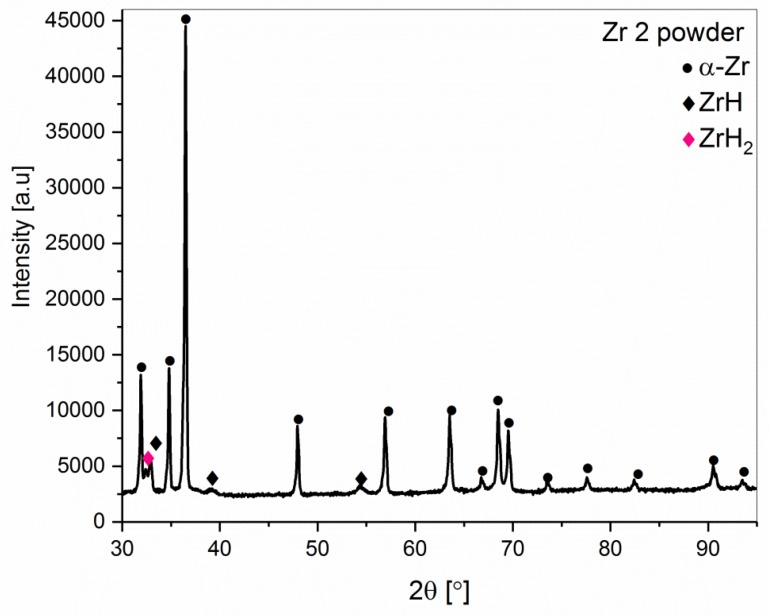
The XRD pattern of the Zr 2 powder (98.8% purity).

The phase constitution of the Zr 1 and Zr 2 powders obtained by XRD powder patterns is shown in Figure 2 and Figure 3. The α-Zr hexagonal close-packed (hcp) structure is observed in both powders. The diffraction lines of the α-Zr correspond to the space group P63/mmc (No. 194) with lattice parameters a = 0.32420 nm and c = 0.51690 nm (No. Card ICDD: 04-008-1477). Additionally, in Zr 2 (Figure 3) powder, hydride phases were detected. The estimated total amount of ZrH_2_ (No. Card ICDD: 04-004-8913) and ZrH (No. Card ICDD: 03-065-6223) was about 15 wt.%.

The accuracy of the XRD techniques depended on the measurement conditions (t = time per step [s] and s = step size [2θ°]) and the crystallographic structure of a given phase. By selecting the measurement conditions appropriately, it was possible to determine even small amounts of a given phase (even below 1%) using the EMPYREAN diffractometer Panalytical, Almelo, The Netherlands.

Additionally, the powder marked Zr 2 contained hydrogen compound impurities, mainly ZrH_2_ and ZrH. The presence of these compounds was related to the process of production of the zirconium powders. The phase compositions of these powders, including a quantitative phase analysis and the changes of the unit cell parameters, are shown in Table 2.

The Zr 1 and Zr 2 powders were pressed into samples at two different pressures, namely 4.0 GPa and 7.8 GPa, at room temperature. The obtained samples were tested by X-ray structural analysis. Comparing the results of the X-ray analysis for the powders and compacts pressed at 4.0 GPa pressure, it should be noted that the phase composition after compression did not change. 

Zr 1 zirconium powder (99.9% purity) pressed at 7.8 GPa also showed no phase change, but for zirconium powders Zr 2 (98.8% purity) the transformation of the α-Zr phase into the ω-Zr phase took place. The content of the ω-Zr phase was estimated to be near 22%. The crystal structure of the ω-Zr phase can be described as the hexagonal structure with space group P6/mmm (No. 191) and lattice parameters: a = 0.50350 nm and c = 0.31410 nm (No. Card ICDD: 04-004-5067). 

The obtained results were different from those of Bridgman [10] and Rabinkin et al. [11], who described the phase transition of pure zirconium at lower pressures, whereas our repeated tests (carried out for 10 samples) did not result in the transformation of the α-Zr phase into ω-Zr for the zirconium powder with 99.9% purity. The presence of hydrides in thee Zr 2 powder clearly contributed to the transformation, which should be linked to the state of stress in the material subjected to compression under high-pressure conditions.

At high pressure conditions (up to 7.8 GPa), in most of materials, the phase transition takes place or the deformation of elementary cells occurs as a result of the increased residual stresses in the material. The deviation of the measured lattice parameters from the theoretical value (%) were obtained as the average of three measurements of the same material.

**Figure 4:**
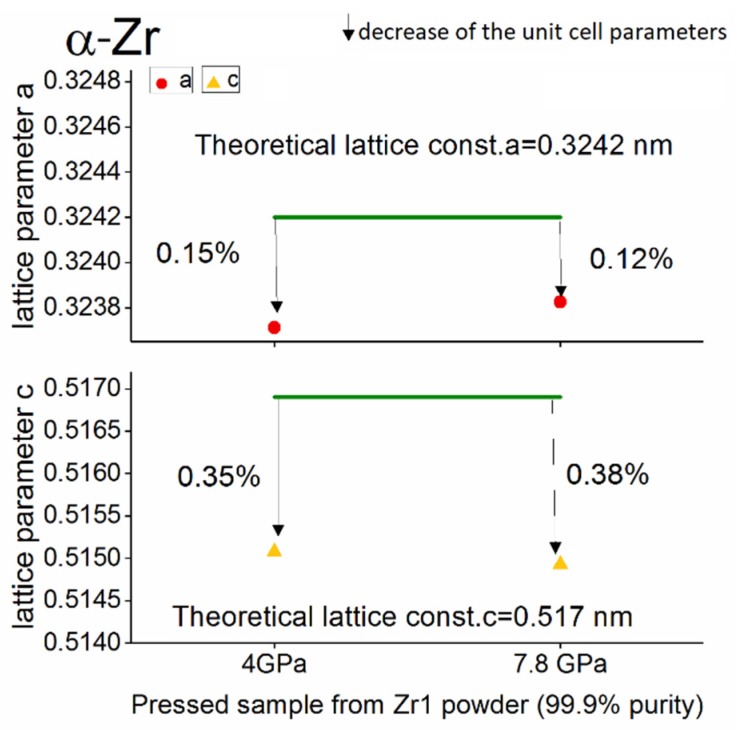
The change of the lattice parameters (in %) for the α-Zr phase in the pressed sample compacted from high purity powder—Zr 1 (99.9%).

**Figure 5:**
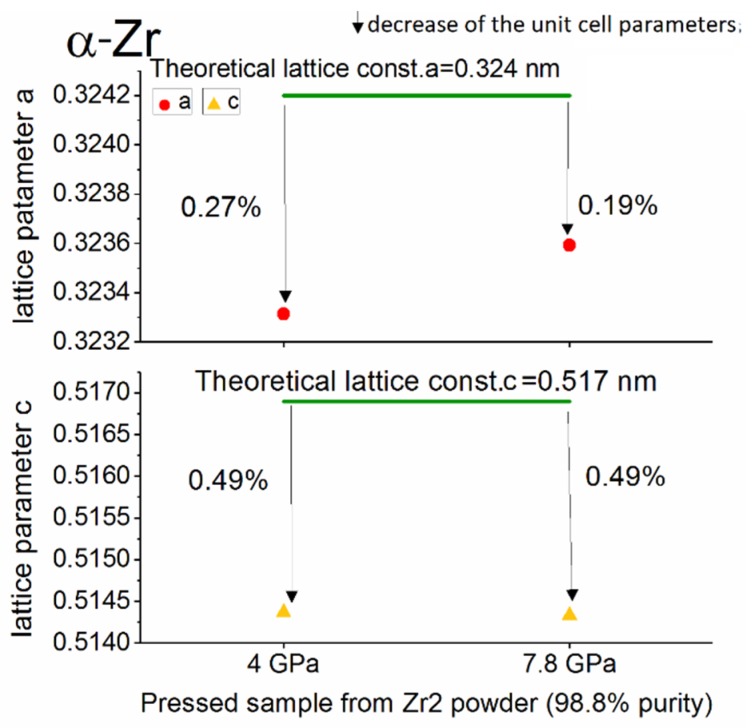
The change of the lattice parameters (in %) for the α-Zr phase in the pressed sample compacted from Zr 2 powder (98.8% purity).

**Figure 6:**
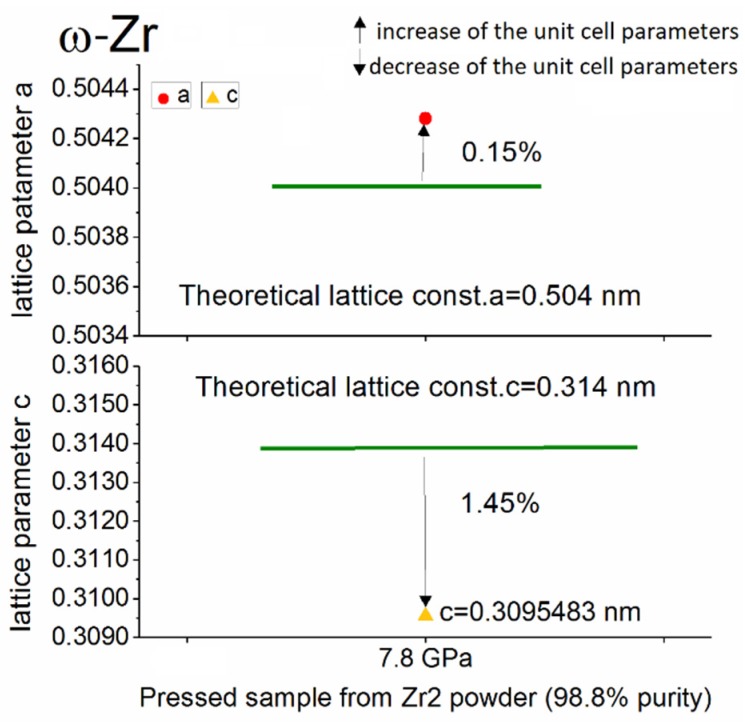
The change of the lattice parameters (in %) for the ω-Zr phase in the pressed sample compacted from Zr 2 powder (98.8% purity).

The results of the structural analysis of the pressed powders for the main phases—α-Zr and ω-Zr—are presented in Figure 4, Figure 5 and Figure 6.

A detailed analysis of the lattice parameters in the samples pressed at different pressure conditions shows that for high-purity powder (Zr 1, 99.9%), the α-Zr phase unit cell parameters did not depend on the applied pressure. Furthermore, both the *a* and *c* units cell parameter changes of the α-Zr form obtained from the Zr 1 powder were lower than those obtained from the 98.8% purity Zr powder (Zr 2). Figure 6 summarises the results of the structural analysis of the ω-Zr phase obtained in the Zr 2 powder at a pressure of 7.8 GPa.

The analysis of this structural data of the ω-Zr phase obtained at 7.8 GPa indicates that the transformation to ω-Zr from α-Zr caused an increase of cell parameter *a* (+0.15%) and significant decrease of the *c* parameter compared to the theoretical one (−1.45%)

**Figure 7:**
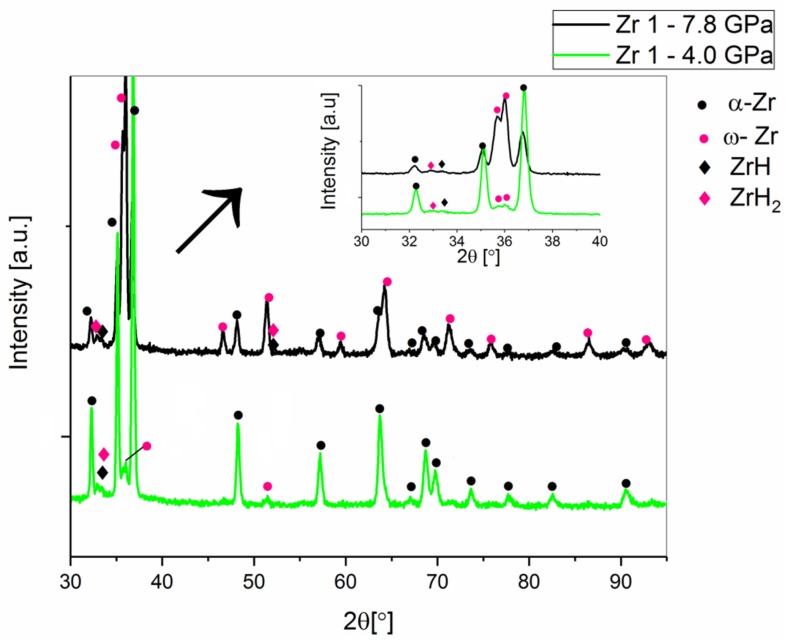
The X-ray diffractograms of the Zr 1 powder sintered at 4.0 GPa and 7.8 GPa, at a temperature of 1273 K.

In Figure 7 and Table 3, the comparative X-ray diffractions of the Zr 1 material sintered at 1273 K under the two different pressures of 4.0 GPa and 7.8 GPa, respectively, are presented.

**Figure 8:**
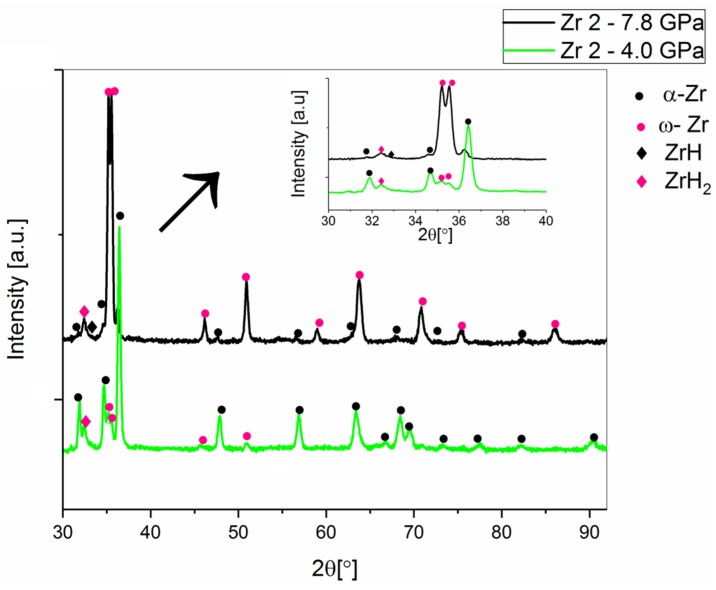
The X-ray diffractograms for sintered Zr 2 powders (98.8% purity), under pressures of 4.0 GPa and 7.8 GPa, sintered at 1473 K.

As a result of the phase transformation during high-pressure sintering at a temperature of 1273 K and a pressure of 4.8 GPa for the Zr 1 powder (99.9% purity), 6 wt.% of the omega Zr phase was detected, whereas at the same temperature, under 7.8 GPa, 64% of the omega phase was determined. The diffraction peaks of hydrides that were not visible for the non-sintered Zr 1 compacts were observed for the sintered materials (Figure 7). Hydrogen is present in both Zr 1 and Zr 2 powders. The information presented in Table 1 shows that Zr 1 zirconium powder contains less hydrogen and the hydrides are not registered by X-ray diffraction. Hydrogen in the atomic form has very limited solubility in the zirconium and zirconium alloys: less than 1 ppm at room temperature, about 80 ppm at 573 K and near 200 ppm at 673 K [21,22]. It can therefore be assumed that the hydrogen in the powders is mainly present as hydrides and not in the atomic form. During high-pressure compacting (7.8 GPa) in the sintering process, the size and the degree of the crystallites of the compacts were increased, hence, the hydrides appeared on the diffractograms of the Zr 1 and Zr 2 sintered materials. In Figure 8 and Table 4, the results for the Zr 2 sintered zirconium powder at 1473 K pressed at 4.0 and 7.8 GPa are shown.

For the Zr 2 powders containing hydrides, the phase transformation of α-Zr into ω-Zr was detected. During sintering at 4.0 GPa, the contribution of ω-Zr was 12%, contrary to the 87% obtained during sintering at the same temperature under a 7.8 GPa pressure. The temperature dependence of the α-Zr to ω-Zr transformation is presented in Table 5. The literature contains information indicating that the decomposition of zirconium hydrides in the heating process takes place at 1073 K in a high vacuum during 30 min [23]. For the sintering HPHT Zr 2 powders, hydride decomposition probably occurred.

The transformation of α-Zr into ω-Zr took place more intensively in conditions of elevated temperatures. This was concluded from the comparison of the transformation data for the Zr samples pressed at room temperature and those pressed at a sintering temperature (Table 3, Table 4, Table 5 and Table 6). However, the difference in the proportion of the ω-Zr phase for the sintered materials at 1273 K and 1473 K was not so significant (Table 5).

### 3.2. Stress Measurements for High-Pressure Compacting and Sintering Zirconium Powders

The stress measurement was performed using the sin^2^ψ X-ray diffraction method. It is based on the angular shift of the Bragg reflection, caused by a change in the d_hkl_-spacing. When the sample was loaded, the lattice spacing changed in accordance with the orientation of the occurring stresses. The dependence between ε(ψ,t) and sin^2^ψ described the fundamental relation of the stress state with the cylindrical symmetry [24,25]. The general equation for the residual stress calculation was given by applying the correct transformations between the diffractometer coordinate system and the investigated specimen:

(1) $$\varepsilon \left(\psi ,t\right)=\frac{d_{\psi }-d_{0}}{d_{0}}=\frac{1+\nu }{E}\left[\sigma_{11}\left(t\right)-\sigma_{33}\left(t\right)\right]\sin^{2}\psi +\frac{1}{E}\left[\sigma_{33}\left(t\right)-2{\nu \sigma }_{11}\left(t\right)\right]$$

where ψ is the tilted angle, d_0_ is the stress-free lattice spacing, d_ψ_ is the lattice spacing measured in the stress sample with the orientation ψ, E is Young’s modulus, ν is Poisson’s ratio, σ_11_ is the stress referred to the component parallel to the investigated sample surface, and σ_33_ corresponds to the parallel component to the cylinder axis of the sample.

**Figure 9:**
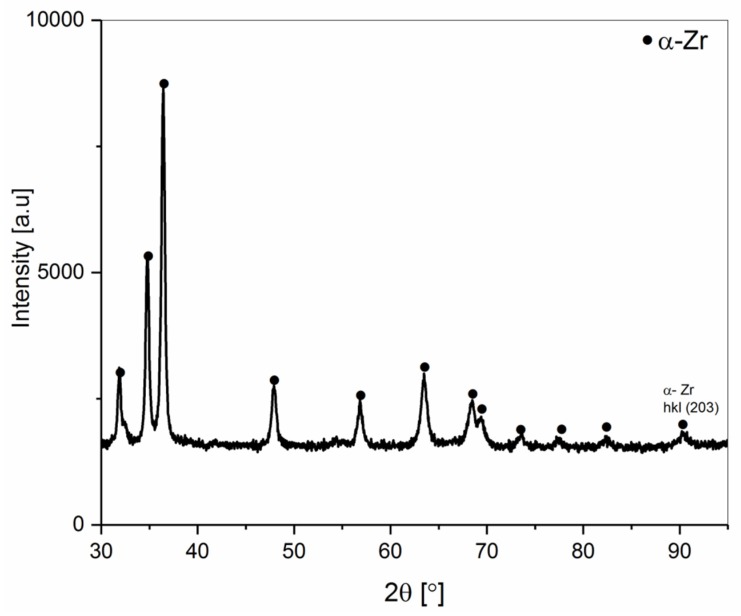
The X-ray diffraction pattern for the test sample obtained from Zr 1 powder with marked peak accepted as a reference point for stress measurement.

The macroscopic residual stresses were measured in the zirconium phase for the hkl (203) diffraction line for the α-Zr phase (peak located at a 90° angle, 2θ) (Figure 9), which is the majority of this phase in the material.

**Figure 10:**
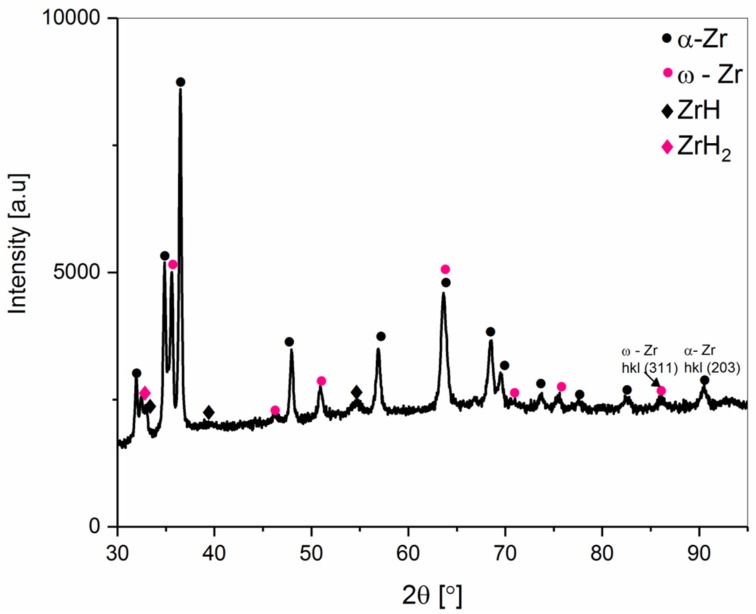
The X-ray diffraction pattern for the compacted Zr 2 powder with the marked peak accepted as a reference point for stress measurement.

In the case of the sample in which the ω-Zr phase appeared, additional residual stress tests were carried out. Residual stresses were measured in the ω-Zr phase characterised by hkl (311)—located for 2θ = 86° (Figure 10).

Table 6 and Table 7 present the results of the residual stress analysis in the high-pressure compacted material. 

According to the results presented in Table 6, higher comprehensive residual stress values were obtained for samples prepared at higher pressures. For the 99.9% pure powders, these values were lower than for the powders with 98.8% purity containing zirconium hydrides. Additionally, the appearance of the omega phase resulted in the formation of strong compressive stresses in the alpha phase, which compensated for the tensile stresses in the omega phase. Table 7 presents the results of the analysis of the residual stresses in the sintered Zr 1 and Zr 2 materials at different sintering temperatures. 

The higher sintering temperature resulted in a decreased comprehensive residual stresses in the ω-Zr phase of the sintered zirconium. It seems that higher levels of stress limited the transformation of the α-Zr phase to the ω-Zr phase.

### 3.3. The ω-Zr Phase Stability at High Temperatures

**Figure 11:**
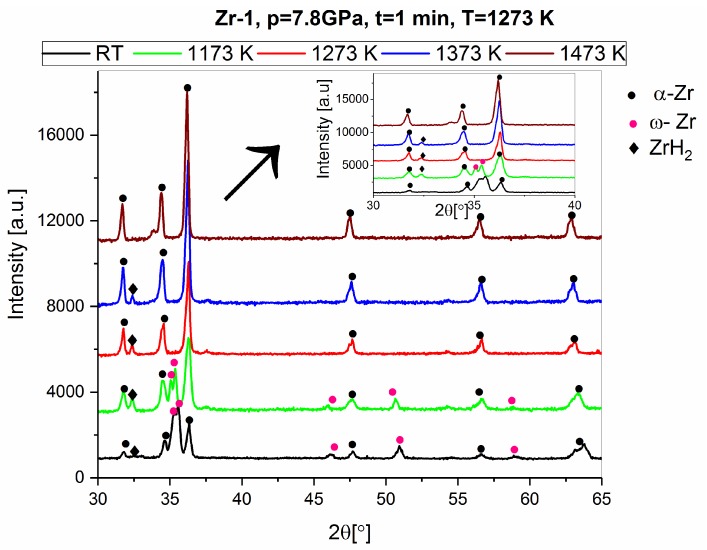
The X-Ray pattern for the heat treatment in the HTK2000 device of the Zr 1 high-pressure material sintered at 1273 K.

In the study presented in this publication, a high-temperature chamber (Anton Paar HTK 2000N) was used for the phase transformation studies during heating at 20 K min^−1^. XRD measurements were performed after the sample was held at a fixed temperature for a period of 10 min. The phase compositions of the materials obtained from the powders of 99.9% purity (Zr 1) during the heating process were determined. The phase changes of the high-pressure sintered Zr 1 material at 1273 K during heating are shown in Figure 11 and in Table 8. 

**Figure 12:**
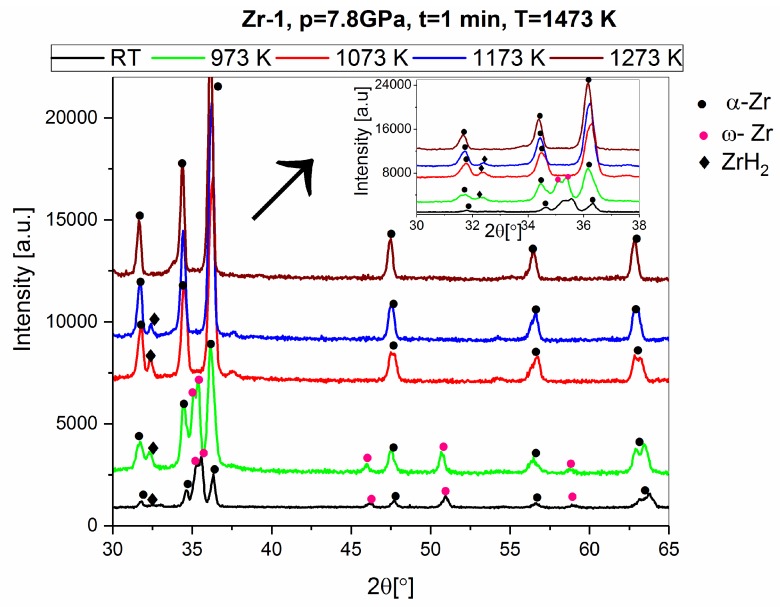
The X-Ray pattern for the heat treatment in the HTK2000 device of the Zr 1 high-pressure material sintered at 1473 K.

The phase changes of the high-pressure sintered Zr 1 material at 1473 K during heating are shown in Figure 12 and Table 9.

In the Zr 1 material sintered at a lower temperature (1273 K), the ω-Zr phase is more stable, and is present in this material up to 1173 K (Table 8). For the Zr 1 sintered material at 1473 K, the ω phase is present up to 973 K. The macrostresses in the sintered material at 1273 K were about −431 MPa, whereas for the sintered material at 1473 K, they were at a level of −333 MPa (Table 7). The results of the ω-Zr phase stability tests are difficult to compare because they were performed by various methods. For example, the dilatometer method was used to study stability [26]. A more direct method was used by Brown et al. [2]. In their study, the composite microstructure of two-phase (α/ω) shocked zirconium was studied in situ during heating (constant heating rate and isothermally) with high-energy X-ray diffraction techniques. The volume fraction of the metastable ω phase was monitored as the reverse phase transformation occurred. The start and finish temperatures were 470 and 550 K, respectively, during heating at a rate of 3 K min^−1^. Isothermal transformation was observed when the shocked material was held at fixed temperatures from 420 to 525 K. 

Undoubtedly, the ω-Zr phase method also influences the transformation process. Brown et al. [2] used zirconium after the shock-wave compression, whereas to obtain the investigated materials, they used the static High Pressure-High Temperature method. There were differences in the state of the stresses and probably in the defect densities of the materials. It can be concluded that the material containing the Zr omega phase obtained by the HPHT method shows higher stability at higher temperatures than zirconium after the shock-wave compression.

## 4. Conclusions

- The pressing of powders under static high-pressure conditions of 7.8 GPa using a Bridgman apparatus led to the transformation of the α-Zr phase into the ω-Zr phase at room temperature. The transformation occurred for zirconium powder of 98.8% purity (with hydrides participation), but did not occur for high purity (99.9%) zirconium powders. After transformation, the ω-Zr content was ~22%.
- The transformation of α-Zr into ω-Zr occurred during the sintering process at 4.0 GPa for both type of zirconium powders (99.9% and 98.8% purity). The amount of ω-Zr phase contents depended on the pressure and the temperature of the HPHT process. The conditions of the transformation process were more dependent on the pressure than on the temperature.
- The highest content of the ω-Zr phase was obtained in the Zr 2 material (98.8% purity with hydrides content) sintered for 1 min at a temperature of 1473 K and a pressure of 7.8 GPa.
- The higher sintering temperature resulted in a decrease of the residual stresses in the ω-Zr phase in the sintered zirconium. The higher levels of stress limited the transformation of the α-Zr phase into the ω-Zr phase.

## Figures and Tables

**Table 1:** Purity and particle sizes of zirconium powders.

Powder Determination	Zr 1	Zr 2
Supplier/supply procedure	BIMOTECH, Poland/in an aqueous suspension	KAMB, Poland/in an aqueous suspension
Purity [%]	99.9	98.8
Particle size by the producer [µm]	60	<45
Measured particle size-median [µm]	12	6
Oxygen content [%]	1.6 ± 0.08	0.75 ± 0.01
Nitrogen content [ppm]	121.0 ± 8.8	446.5 ± 18.3
Hydrogen content [%]	0.155 ± 0.02	0.381 ± 0.002

**Table 2:** The phase compositions of the Zr 1 (99.9% purity) and Zr 2 (98.8% purity) powders.

Powder Symbol	Phase Composition	Unit Cell Parameters		Phase Content [wt.%]	Agreement Indices *
		From ICCD Data Base [nm]	Fitting by Rietveld Method [nm]		
Zr 1	α-Zr	a = 0.3242 c = 0.5169	a = 0.3234052 c = 0.5147363	100	R_exp_ = 2.13%R_p_ = 2.07% R_wp_ = 2.92%
Zr 2	α-Zr	a = 0.3242 c = 0.5169	a = 0.3229767 c = 0.5143974	85	R_exp_ = 2.10% R_p_ = 2.04% R_wp_ = 2.82%
	ZrH	a = 0.4586 c = 4.948	a = 0.4594012 c = 0.4946331	10	
	ZrH_2_	a = 0.5000 c = 0.4440	a = 0.500129 c = 0.4462180	5	

* R_exp_—expected R factor, R_p_—profile R factor, R_wp_—weighted profile R-factor.

**Table 3:** The qualitative and quantitative composition data of sintered Zr 1 powder (sintering conditions: T = 1273 K, t = 1 min).

Pressure of the Sintering 4.0 GPa			Pressure of the Sintering 7.8 GPa		
Phase Constitution	wt.%	Agreement Indices *	Phase Constituion	wt.%	Agreement Indices
α-Zr	91	R_exp_ = 3.13% R_p_ = 3.01% R_wp_ = 4.69%	α-Zr	33	R_exp_ = 3.13% R_p_ = 2.52% R_wp_ = 3.73%
ω-Zr	6		ω-Zr	64	
ZrH	2.5		ZrH	2	
ZrH_2_	0.5		ZrH_2_	1	

* R_exp_—expected R factor, R_p_—profile R factor, R_wp_—weighted profile R-factor.

**Table 4:** The qualitative and quantitative composition data of sintered Zr 2 powder (sintering conditions: T = 1473 K, t = 1 min)**.**

Pressure 4.0 GPa		Pressure 7.8 GPa	
Phase Type	wt.%	Phase Type	wt.%
α-Zr	79	α-Zr	7
ω-Zr	12	ω-Zr	87
ZrH	-	ZrH	1
ZrH_2_	9	ZrH_2_	5

**Table 5:** The phase composition of the Zr 1 and Zr 2 sintered materials in 7.8 GPa depending on the temperature of the sintering process.

Phase Type	Zr 1		Zr 2	
	T = 1273 K, t = 1 min, wt.%	T = 1473 K, t = 1 min, wt.%	T = 1273 K, t = 1 min, wt.%	T = 1473 K, t = 1 min, wt.%
α-Zr	33	38	12	7
ω-Zr	64	60	79	87
ZrH	1	-	-	1
ZrH_2_	2	2	9	5

**Table 6:** The residual stresses in Zr 1 and Zr 2 high-pressure compacted powders.

Powder/Pressure of Compacting	Zr 1		Zr 2	
	4.0 GPa	7.8 GPa	4.0 GPa	7.8 GPa
α-Zr_(203)_	−133 ± 24 MPa	−159 ± 15 MPa	101 ± 9 MPa	−567 ± 52 MPa
ω-Zr_(311)_	lack of ω-Zr phase	lack of ω-Zr phase	lack of ω-Zr phase	223 ± 72 MPa

**Table 7:** The residual macrostresses in the tested materials for various sintering temperatures at a 7.8 GPa pressure.

Phase/Sample Sintering Parameters	Zr 1		Zr 2	
	T = 1273 K, t = 1 min [MPa]	T = 1473 K, t = 1 min [MPa]	T = 1273 K, t = 1 min [MPa]	T = 1473 K, t =1 min [MPa]
α-Zr_(203)_	−37 ± 21	−269 ± 53	Too low amount of α-Zr phase for calculations	Too low amount of α-Zr phase for calculations
ω-Zr_(311)_	−431 ± 43	−333 ± 30	−307 ± 25	−214 ± 21

**Table 8:** The qualitative and quantitative phase composition (wt.%) after heat treatment in the HTK2000 device of the Zr 1 material high-pressure sintered at 1273 K.

Sample Sintering Parameters	Zr 1, 7.8 GPa, T = 1273 K, t = 1 min				
Phase Type/Temperature in the HTK2000 Device	RT	1173 K	1273 K	1373 K	1473 K
α-Zr	33	70	95	98	100
ω-Zr	64	25	-	-	-
ZrH	2	-	-	-	-
ZrH_2_	1	5	5	2	-

**Table 9:** The qualitative and quantitative phase composition (wt.%) after heat treatment in the HTK2000 device of the Zr 1 material high-pressure sintered at 1473 K.

Sample Sintering Parameters	Zr 1, 7.8 GPa, T = 1473 K, t = 1 min				
Phase Type/Temperature in the HTK2000 Device	RT	973 K	1073 K	1173 K	1273 K
α-Zr	38	64	96	98	100
ω-Zr	60	34	-	-	-
ZrH	-	-	-	-	-
ZrH_2_	2	2	4	2	-
